# Complete Genome Sequences of Four Bacteria Isolated from the Gut of a Spiny Ant (*Polyrhachis lamellidens*)

**DOI:** 10.1128/mra.00333-22

**Published:** 2022-06-06

**Authors:** Sora Ishikawa, Muyang Huang, Atsuki Tomita, Yu Kurihara, Riki Watanabe, Hironori Iwai, Kazuharu Arakawa

**Affiliations:** a Institute for Advanced Biosciences, Keio University, Tsuruoka, Yamagata, Japan; b Systems Biology Program, Graduate School of Media and Governance, Keio University, Fujisawa, Kanagawa, Japan; c Faculty of Environment and Information Studies, Keio University, Fujisawa, Kanagawa, Japan; Indiana University, Bloomington

## Abstract

We isolated and sequenced the complete genomes of four bacteria from the gut contents of a spiny ant (*Polyrhachis lamellidens*) sampled from a primeval beech forest in Yamagata Prefecture, Japan. The isolates belong to the genera *Tsukamurella*, *Enterococcus*, *Lysinibacillus*, and *Streptomyces* and provide insights into the functional roles of microbiomes of ants.

## ANNOUNCEMENT

Bacterial communities are deeply involved in symbiotic interactions in ants (1), but the detailed functional associations with the host species remain largely unknown. Here, we present the isolation and sequencing of four bacteria that were isolated from the gut contents of a spiny ant (Polyrhachis lamellidens) sampled from a primeval beech forest in Yamagata Prefecture, Japan. The isolated bacteria belong to the genera *Tsukamurella*, *Lysinibacillus*, *Enterococcus*, and *Streptomyces*, all of which are closely related to previously detected microbes in ant gamergates and males (2). Ant species, such as leafcutter ants, can associate with plant bacterial community members of the *Tsukamurella* genus (3). Although studies suggest the significance of *Tsukamurella* in a bacterial community, its effects and functions in a gut microbiome are largely unknown.

We first isolated the microbes by dissecting the midgut of three workers of P. lamellidens, and the supernatants after homogenization and centrifugation were inoculated on LB agar medium and incubated at 37°C overnight. Each ant was thoroughly washed with phosphate-buffered saline (PBS) and sterilized equipment was used to minimize contamination. Several single colonies were picked and incubated again on separate LB agar plates at 37°C overnight. Proliferated samples were collected the next day and stored at −20°C. Subsequently, genomic DNA was extracted from thawed samples in LB liquid medium with Genomic-tip 20/G (Qiagen), following the manufacturer’s protocol. Long-read sequencing libraries were prepared and multiplexed using the rapid barcoding kit (SQK-RAB0004; Oxford Nanopore Technologies) and then were sequenced with FLO-MIN106 flow cell, base called with Guppy version 5.0.12 (super-accurate mode), demultiplexed and adapter trimmed in a on a GridION X5 device (GridION software release 21.05.25; Oxford Nanopore Technologies), and checked for quality with NanoPlot (4). An Illumina sequencing library for error correction was prepared using a KAPA HyperPlus kit (Kapa Biosystems), and the library was sequenced on a NextSeq 500 sequencer (Illumina) using the 75-cycle high-output mode with single ends. The long reads obtained were subsampled to yield around 50- to 100-fold coverage and were assembled using Canu version 2.2 (5). All assemblies generated a single assembled chromosome, which was manually circularized by deleting the overlapping ends. The assembled chromosome was further error corrected using all unfiltered Illumina reads with Pilon version 1.2.4 (6), which was repeated until the completeness statistics were saturated. The assembly was checked for completeness with CheckM (7). Subsequently, the assembly was annotated and circular genomes were rotated so that the *dnaA* gene was at the start position using DDBJ Fast Annotation and Submission Tool (DFAST) (8). Sequence accession numbers and sequenced read, genome assembly, and annotation statistics are listed in Table 1. Default parameters were used except where otherwise noted.

**TABLE 1:** Accession numbers and sequencing statistics for the isolated strains

Bacterial species	Isolate	DDBJ accession no.	BioProject accession no.	SRA accession no.		Total size of Nanopore reads (Mb)	Nanopore read *N*_50_ (bp)	Total size of Illumina reads (Mb)	No. of contigs	Genome assembly size (bp)	No. of coding sequences	G+C content (%)	No. of rRNAs	No. of tRNAs
				Nanopore reads	Illumina reads									
*Tsukamurella* sp.	PLM1	AP025688	PRJNA820548	SRR18513073	SRR18513072	116.19	29,422	1,836.33	1	4,391,067	4,809	70.7	6	57
*Lysinibacillus* sp.	PLM2	AP025689	PRJNA820550	SRR18513094	SRR18513093	283.42	14,190	2,577.20	1	4,015,857	3,945	35.0	34	106
*Enterococcus* sp.	PLM3	AP025690	PRJNA820552	SRR18513233	SRR18513232	298.00	8,417	2,409.01	1	2,859,161	2,667	37.5	12	62
*Streptomyces* sp.	PLM4	AP025692	PRJNA820554	SRR18513231	SRR18513230	331.76	12,063	1,867.82	1	7,000,089	6,128	73.5	21	76

According to the annotation results, we found various proteins suggesting intricate environmental interactions, such as the protein involved in the control of the pheromone-responding pAD1 replicon (9) and several antibiotic resistance proteins. The availability of the complete genomes of these ant symbionts should contribute to the study of ant-microbe ecological interactions.

### Data availability.

The genome sequences reported here were deposited in DDBJ, and the raw reads were deposited in the Sequence Read Archive (SRA), with the accession numbers listed in Table 1.
